# Efficacy of a Gluten-Free Diet in the Gilles de la Tourette Syndrome: A Pilot Study

**DOI:** 10.3390/nu10050573

**Published:** 2018-05-07

**Authors:** Luis Rodrigo, Nuria Álvarez, Enrique Fernández-Bustillo, Javier Salas-Puig, Marcos Huerta, Carlos Hernández-Lahoz

**Affiliations:** 1 Gastroenterology Unit, Hospital Universitario Central de Asturias (HUCA), Avda. de Roma s/n, 33011 Oviedo, Spain; nuriaalvarezh@gmail.com; 2 Technical Department, Hospital Universitario Central de Asturias (HUCA), Avda. de Roma s/n, 33011 Oviedo, Spain; bustillo.e@telefonica.net; 3 Neurology Service, Hospital del Valle de Hebrón, Paseo del Valle de Hebrón 119, 08035 Barcelona, Spain; jsalasp@meditex.es; 4 Psychiatry Service, Mental Health Center, Pedro Pablo 42, 33209 Gijón, Spain; marcoshuerta47@gmail.com; 5 Neurology Service, Hospital Universitario Central de Asturias (HUCA), Avda. de Roma s/n, 33011 Oviedo, Spain; carloshlahoz@gmail.com

**Keywords:** Gilles de la Tourette syndrome (GTS), children and adults, motor and vocal/phonic tics, obsessive-compulsive disorder (OCD), non-coeliac gluten sensitivity (NCGS), gluten-free diet, one-year adherence

## Abstract

The Gilles de la Tourette syndrome (GTS) and Non-Coeliac Gluten Sensitivity (NCGS) may be associated. We analyse the efficacy of a gluten-free diet (GFD) in 29 patients with GTS (23 children; six adults) in a prospective pilot study. All of them followed a GFD for one year. The Yale Global Tics Severity Scale (YGTSS), the Yale-Brown Obsessive-Compulsive Scale—Self Report (Y-BOCS) or the Children’s Yale-Brown Obsessive-Compulsive Scale—Self Report (CY-BOCS), and the Cavanna’s Quality of Life Questionnaire applied to GTS (GTS-QOL) were compared before and after the GFD; 74% of children and 50% of adults were males, not significant (NS). At the beginning of the study, 69% of children and 100% of adults had associated obsessive-compulsive disorder (OCD) (NS). At baseline, the YGTSS scores were 55.0 ± 17.5 (children) and 55.8 ± 19.8 (adults) (NS), the Y-BOCS/CY-BOCS scores were 15.3, (standard deviation (SD) = 12.3) (children) and 26.8 (9.2) (adults) (*p* = 0.043), and the GTS-QOL scores were 42.8 ± 18.5 (children) and 64 ± 7.9 (adults) (*p* = 0.000). NCGS was frequent in both groups, with headaches reported by 47.0% of children and 83.6% of adults (*p* = 0.001). After one year on a GFD there was a marked reduction in measures of tics (YGTSS) (*p* = 0.001), and the intensity and frequency of OCD (Y-BOCS/CY-BOCS) (*p* = 0.001), along with improved generic quality of life (*p* = 0.001) in children and adults. In conclusion, a GFD maintained for one year in GTS patients led to a marked reduction in tics and OCD both in children and adults.

## 1. Introduction

The Gilles de la Tourette syndrome (GTS) is a chronic neuropsychiatric process of unknown cause. It is characterised by the presence of multiple motor tics and at least one vocal or phonic tic. Both types of tic are usually intermittent, although not necessarily concurrently. They are of variable frequency, with periods of intensification and remission, persisting for more than a year, from the appearance of the first tic [1].

This disorder begins in childhood or adolescence before the age of 18 years [1]. Tic severity worsens throughout childhood and for most patients, the worst ever period of tics occurs between 8 and 12 years of age [2,3]. Although up to 80% of patients with GTS have a significant tic decrease during adolescence, and by age 18 years tic intensity and frequency have decreased to such an extent that the person no longer experiences any impairment from their tics, objective ratings indicate that up to 90% of adults continue to exhibit mild tics, although they may occasionally pass unnoticed [3,4]. Its prevalence in school-age children worldwide is around 1%, with a clear predominance in males compared with females on average (3:1). The GTS may be associated with other comorbidities in up to 90% of cases, including obsessive-compulsive disorder (OCD) and those related with attention-deficit/hyperactivity disorder (ADHD) [5]. When comorbid OCD debuts during childhood, it tends to remit in adulthood in only about 40% of cases. It also can develop during adolescence or early adulthood [3,6].

Non-coeliac gluten sensitivity (NCGS) was first described in 1980 [7], but it was classified as part of the spectrum of gluten-related disorders, which also includes coeliac disease (CD) and wheat allergy (WA), until being recognised as a separate clinical entity in 2010. The NCGS is the most frequent of these and is estimated to occur at a prevalence as high as 13% in the general population [8,9].

The clinical presentations of NCGS are varied and overlap with those of CD. It is diagnosed through the prior exclusion of CD, because the serological and histological markers of gluten are usually negative and show a positive response to the withdrawal of gluten from the diet [10,11]. The extra-intestinal symptoms may be the only manifestations of the NCGS, affecting the skin and the musculoskeletal and nervous systems in general [12]. All the associated symptoms improve notably, even disappearing with prolonged adherence to a gluten-free diet (GFD), in a similar manner to what occurs in coeliac patients [13].

The spectrum of neurological processes associated with gluten has progressively broadened in recent years [14,15,16]. We might expect that the neurological symptoms in certain patients with GTS would maintain a certain relation with the presence of a previously unknown associated NCGS. For this reason, the GFD could have a beneficial effect on their general symptomatology, including neurological symptoms. At present, there is little evidence of its utility in these patients and only isolated cases have provided evidence of the efficacy of a GFD, showing that it could be beneficial [17,18,19], as has been reported in patients with autism; in these cases, milk casein was also eliminated from the diet of many of them [20]. Its long-term efficacy has been described in one isolated case of GTS treated with GFD for 3 years, whose neurological and general symptomatology completely recovered. Currently, no controlled studies are available of series of patients. A recent systematic review of the literature on the influence of different dietary interventions in patients with GTS found nine articles and one book chapter, none of which included isolated comparative or inter-group studies [21].

The aim of the current study was to analyse and evaluate the efficacy of the GFD, followed for a year by a series of child and adult patients with GTS. The evolution of the neurological and other symptoms associated with NCGS, and the changes observed in their quality of life were evaluated, enabling the comparison of existing clinical aspects before starting the GFD and after 1 year of adherence to it.

## 2. Materials and Methods

We carried out a prospective pilot study at the national level in Spain of patients diagnosed with GTS to evaluate the efficacy of following a GFD in children and adults. Participants were recruited as voluntary in the study through the online invitation from the National Forum for Tourette’s Syndrome. Adults or children’s parents gave their written informed consent before they participated in the study. Children are considered to be those younger than 14 years before inclusion in the study. A fundamental inclusion criterion for all patients was that they exhibited motor tics and at least one vocal/phonic tic that had lasted for more than one year.

Thirty-four consecutive patients with GTS began the study, comprising 26 children and eight adults diagnosed, evaluated and followed up various specialists (paediatricians, neurologists, general practitioners, psychiatrists and psychologists). Five patients (three children and two adults) were withdrawn, three due to prolonged interruptions of the diet (one child and two adults) and two children due to voluntary abandonment of the diet. The final sample therefore comprised 29 patients (23 children and six adults).

The children’s parents and the adults voluntarily agreed to take part in the study, having been provided with detailed information about the characteristics of the GFD and control criteria. The study was carried out in accordance with Good Clinical Practice, in accordance with the Personal Data Protection and Confidentiality Law, thereby maintaining the anonymity of patients at all times. This study received the ethical approval for the Committee of the Hospital Universitario Central de Asturias (HUCA) with the ethical code number 6265481/16.

Patients were recommended to follow a strict and permanent GFD, as was explained in detail to the parents and adults, and to avoid all types of contamination, for a minimum period of adherence of one year. The observed differences in clinical evolution were compared, and the findings and data collected during the period before starting the GFD and at the end of the year adhering to the diet.

Upon joining the study, patients were asked to undergo a wide range of blood tests, including a complete blood count, general biochemistry, levels of serum anti-tissue transglutaminase antibodies (TGt), the CD genetic markers Human Leukocyte Antigen-DQ2 and Human Leukocyte Antigen-DQ8 (HLA-DQ2 and HLA-DQ8), and serum levels of thyroid hormone and of 25(OH)-Vit. D.

Four questionnaires were administered upon commencement and after 1 year on the GFD. Two of these covered different aspects of GTS, with respect to tics and to OCD. The other two addressed quality of life: a generic one and another one specific to GTS. All the questionnaires were supervised by the parents of the children or filled in by the adults themselves. Information was collected about the principal symptoms related to the patients’ neurological characteristics and the signs and symptoms associated with the presence of NCGS.

Our team reviewed all the questionnaires received to ensure that they were complete and contained no contradictory responses. Contact by regular e-mails, telephone calls and/or face-to-face meetings was maintained throughout the study with the parents of the children and with the adults themselves to resolve queries and check the data.

### 2.1. Questionnaires Used in the Study

#### 2.1.1. Questionnaire to Assess the Severity of Tics Using the Yale Global Tics Severity Scale (YGTSS)

The YGTSS is a widely used instrument for evaluating the intensity and clinical severity of tics in patients with GTS. A variety of tics are enumerated and scored to derive three subscales: motor tics, vocal/phonic tics, and the impairment caused by the tics. For each tics scale, the mean number, total frequency, intensity, complexity and interference are scored between 0 (no affectation) and 5 (maximum affectation). The highest possible overall score for motor tics is 25 and for phonic tics is 25. The score for the impairment arising varies between 0 (none) and 50 (maximum). The total score for the YGTSS is obtained by summing the results obtained from the three subscales and has a maximum value of 100. We used the validated Spanish version of the YGTSS [22,23].

#### 2.1.2. Questionnaires for Evaluating OCD Using the Yale-Brown Obsessive-Compulsive Scale—Self Report (Y-BOCS) and the Children’s Yale-Brown Obsessive-Compulsive Scale—Self Report (CY-BOCS)

The Y-BOCS and the CY-BOCS are used to evaluate obsessions and compulsions in children and adults, respectively. They comprise ten items, scored from 0 (no symptoms) to 4 (severe symptoms), to evaluate three components: (a) Obsessions: obtained as the sum of the first five items: Time occupied by the main obsession, its interference in daily life, the distress caused, the resistance against them, and the control over them; (b) Compulsions: comprising the latter five items, which likewise evaluate the time, interference, distress, resistance and control of the principal compulsion presented by the individual; (c) Overall evaluation of obsessions and compulsions: obtained as the sum of the two previous measures. The overall score varies between 0 (minimum) and 40 (maximum). We used the validated Spanish version [24,25].

#### 2.1.3. EuroQol-5D (EQ-5D) Generic Quality of Life Questionnaire

The EQ-5D is a generic instrument for evaluating a person’s state of general health. It analyses five dimensions (mobility, self-care, usual activities, pain/discomfort, anxiety/depression), each scored on a scale of 1–3, representing best and worst health. The final evaluation includes a summary index, whose maximum value is 1 (indicating a state of full health). It also includes a visual analogue scale (VAS) from 0 to 100, where a value of 100 represents the best imaginable health state. We used the validated Spanish version [26].

#### 2.1.4. Cavanna’s Quality of Life Questionnaire Applied to GTS (GTS-QOL)

This is a specific instrument for evaluating the quality of life of patients with GTS. It comprises 27 items covering six dimensions, each scored from 0 (minimum possible value) to four (maximum possible value): cognitive (eight items); psychological (six items); obsessive-compulsive (four items); physical (three items); coprophenomena (three items); activities of daily living (three items). The results from the six dimensions are evaluated by summing the scores of all the items and, for ease of interpretation, transforming the total to give a value between 0 and 108. It also includes a VAS, scored from 0 to 100, for which the maximum value represents the best possible health. The validated scale in English was administered in Spanish [27].

### 2.2. Evaluation of Other Neurological Characteristics

Other data of GTS were collected at the time of inclusion, including age of onset and duration of the different symptoms of the disease, number of family members affected, and types of medication consumed. The changes that had occurred with respect to the various aspects under investigation by the end of the year on the GFD were analysed.

### 2.3. Evaluation of NCGS Symptoms

The signs and symptoms related to the NCGS were evaluated with a questionnaire comprising several items with a variable number of possible responses. Clinical characteristics of NCGS were collected at the time of inclusion, along with information about family background, analytical results and complementary tests and their evolution after the GFD. A group of questions designed to evaluate the different symptoms distributed by organs and apparatus, each with a variable number of possible responses, and a general score between 0 (absence of symptoms) and 3 (maximum possible intensity) was included.

### 2.4. Evaluation of GFD Compliance

The evaluation and follow up of the diet compliance were carried out through questionnaires filled in by the patients (in the case of adults) or their parents (in the case of children) and regular contact by telephone, e-mail or, in some cases, face-to-face consultation.

### 2.5. Audio-Visual Monitoring Evaluation

For the assessment of tics and following the recommendations of the European clinical guidelines for Tourette Syndrome and other tic disorders, we ask our patients for some audiovisual recordings, reflecting spontaneous situations of everyday life [3]. The parents of one of our patients gave their written informed consent to attach a demonstration video of their child’s evolution for scientific purposes in this study, whose details are specified in the Supplementary Material Section.

### 2.6. Statistical Methods

Data were analysed with SPSS (v15.0 for Windows; SPSS Inc., Chicago, IL, USA). Descriptive analyses were used to characterize the study population. Categorical variables were expressed as absolute frequencies and percentages. Quantitative variables were expressed as the mean or median, if normally or non-normally distributed, respectively, and were compared within and between groups using Student’s two-tailed independent samples *t*-test. To compare the proportions (frequencies) of qualitative variables between the groups, contingency test methods (chi-squared (*χ*^2^) or Fisher’s exact tests) were used, as appropriate. Student’s *t*-test and Mann-Whitney were used for independent (unpaired) samples (children vs. adults). When the number of observations of any of the groups compared was small, always the parametric tests (Student) were used; otherwise, the tests used were Mann-Whitney and Wilcoxon. When the data were quantitative of low rank (for example, scores from 1 to 5) nonparametric tests were always used. For samples of repeated measurements, the McNemar test has been used. The paired Wilcoxon signed-rank test was used to compare differences in the medians of continuous non-parametric variables. The tests used and the variables of the groups in which they have been applied are specified at the beginning of the foot of each table. In all cases, a value of *p* < 0.05 was considered to indicate a statistically significant difference.

## 3. Results

### 3.1. Baseline Demographic Characteristics of Children and Adults

Comparing the baseline characteristics of child and adult patients included in the study, showed that 74% of the children and 50% of the adults were male (NS). 69% of children and 100% of adults presented an associated OCD (NS). ADHD was present in 52.2% of children and 66.2% of adults (NS).

The total tic score, as measured by the YGTSS questionnaire, was similar in the two groups (NS). Conversely, the total OCD score assessed with the Y-BOCS/CY-BOCS questionnaires, was lower in the children 15.3 (12.3) than in the adults 26.8 (9.2) (*p* = 0.043). The generic quality of life score at the beginning of the study was similar in the two groups. However, GTS-specific quality of life was lower in the children 42.8 (18.5) than in the adults 64.0 (7.9) (*p* = 0.000). There was no difference in the consumption of medication between the groups, either overall or with respect to Non-Steroidal Anti-Inflammatory Drugs (NSAIDs). However, many fewer children than adults took psychotropics (34.8% vs. 100%) (*p* = 0.006) (Table 1).

### 3.2. Baseline Characteristics of the Symptoms and Signs of Gluten Sensitivity in Children and Adults

Upon entering the study, a series of symptoms and signs related to the presence of NCGS, which were present in the individual participants, were compared between the two groups. No significant differences were found for any characteristics, except for the presence of headaches and/or migraines, which were more common in the adults than the children (83.3% vs. 47.8%) (*p* = 0.1), and of behavioural disorders, which were also more common in adults (100% vs. 95.6%) (*p* = 0.001) (Table 2).

### 3.3. Evolution of Neurological Symptoms and Quality of Life after 1 Year of a GFD

After one year of following a GFD, the improvement in neurological symptoms was very striking, with a significant reduction in tics and OCD in both children and adults (*p* = 0.001). The same occurred with the improvement found in the generic and specific quality of life (*p* = 0.001), whereby there was no difference between the children and adults. This translated into a reduction in the consumption of medication in both groups, the effect being very pronounced in children (*p* = 0.001) but more moderate in adults (*p* = 0.072); the reduction among the adults was mainly a consequence of a decrease in the consumption of psychotropics (*p* = 0.071) (Table 3).

### 3.4. Evolution after 1 Year of a GFD of the Various Components of Motor and Phonic Tics, Obsessions and Compulsions

The evolution of the different components of the motor and vocal/phonic tics, and of OCD, was assessed, comparing the results obtained before beginning and a year after following the GFD. With respect to the evolution of the characteristics of the motor tics, we found a notable decrease in their various components (number, intensity, frequency, complexity and interference), that was more significant in children (*p* = 0.000) than in adults (*p* = 0.027). The evaluation of the vocal/phonic tics revealed a reduction in their principal characteristics in both groups, again being more pronounced in children (*p* = 0.001) than in adults (*p* = 0.028). This was maintained with an identical significance when jointly evaluating the motor and vocal/phonic tics, this improvement being somewhat smaller in the adults than in the children. The evolution of the disability associated to the motor tics was significantly reduced in the children (*p* = 0.000), but less so in the adults (*p* = 0.059). Likewise, a clear improvement in the evolution of the disability related to the vocal/phonic tics was confirmed, the effect being more significant in the children (*p* = 0.001) than in the adults (*p* = 0.041). The decrease in the overall degree of disability along with the motor and vocal/phonic tics was significant in both groups, though slightly higher in the children (*p* = 0.000) than in the adults (*p* = 0.027). Equally, after one year on the GFD the various components of the obsessions (time, interference, distress, resistance and control) were significantly reduced, which was somewhat more marked in children (*p* = 0.001) than in adults (*p* = 0.028). The same components of the compulsions after a year on a GFD confirmed a significant improvement in both groups, again being slightly greater in children (*p* = 0.008) than in adults (*p* = 0.027) (Table 4).

### 3.5. Evolution of Symptoms of Non-Coeliac Gluten Sensitivity after 1 Year of a GFD

The evolution of the symptoms associated with NCGS after one year on a GFD was also very favourable. The number and intensity of upper airway infections were significantly reduced in both groups, though more notably in the children (*p* = 0.000) than in the adults (*p* = 0.068). The same pattern was found for the lower airway, with children showing a more significant reduction (*p* = 0.001) than the adults (*p* = 0.180). Conversely, no significant differences were found in the number of associated allergies. Fewer episodes of headaches/migraines were observed, the effect being slightly more significant in children (*p* = 0.013) than in adults (*p* = 0.068). Infectious or inflammatory oral processes were notably reduced in children (*p* = 0.000) but were unchanged in adults (*p* = 0.104). Musculoskeletal affectation decreased significantly in children (*p* = 0.002) and slightly less significantly in adults (*p* = 0.042). Associated dermatitis also decreased strikingly in children (*p* = 0.002), more significantly than in the adults (*p* = 0.058). Anaemia and iron deficiency improved notably in children (*p* = 0.004) but was unchanged in adults (*p* = 0.131). Likewise, sleep disorders reduced significantly in the children (*p* = 0.000) and in a smaller proportion of adults (*p* = 0.046). No significant changes in urinary disorders were noted in children (*p* = 0.082) or adults (*p* = 0.109). Behavioural disorders decreased significantly in children (*p* = 0.000) and to a lesser degree in adults (*p* = 0.028). The improvement achieved in the dietary disorders was more evident in children (*p* = 0.000) than in adults (*p* = 0.027), as was the case for the improvement in intestinal habit, which was greater in children (*p* = 0.001) than in adults (*p* = 0.075) (Table 5).

## 4. Discussion

At the beginning of the study, the presence of motor and vocal/phonic tics was similar in children and adults. The generic quality of life was similar in the two groups; however, specifically GTS-related quality of life was worse in children than in adults. Nevertheless, the adults were taking a higher proportion of psychotropics than the children, with significant differences. Our results coincide with those of other authors, in the sense that during childhood the intensity and frequency of tics are usually higher in children than in adults, while OCD usually predominates in adults compared with children, which accounts for the more widespread consumption of psychotropics among adults than children [2,28,29].

People with NCGS usually exhibit a variety of associated symptoms such as headaches or migraines, “brain fog”, fatigue, fibromyalgia, joint and muscle pain, leg or arm numbness, tingling of the extremities, dermatitis (eczema or skin rash), allergies, atopic disorders, depression, anxiety, anaemia, iron-deficiency anaemia, folate deficiency, asthma, rhinitis, eating disorders, or autoimmune diseases. Among the extra-intestinal manifestations, NCGS has been implicated in some neuropsychiatric disorders, such as schizophrenia, autism, peripheral neuropathy, ataxia, ADHD, mood swings, sensory symptoms, disturbed sleep patterns, and hallucinations (“gluten psychosis”) [9,16,30,31,32,33,34,35].

Many coeliac patients or those with undiagnosed NCGS underestimate their multiple and frequent discomfort from digestive and more general causes because they have grown accustomed to living with a state of chronic poor health as though it were normal. They are only able to recognise that they really did have symptoms related to the consumption of gluten when they start the GFD and the improvement becomes obvious [36,37].

The disproportionately common occurrence in patients with GTS of immunologically determined illnesses, such as allergic processes, rhinitis, asthma, dermatitis and conjunctivitis, frequently with raised IgE and a positive family history of autoimmune diseases has been reported. Likewise, the presence of migraines, autistic spectrum disorders, anxiety, depression, sleep disorders, behavioural problems and hallucinations have frequently been noted [38,39,40].

At the beginning of our study, various symptoms and signs associated with NCGS were present in similar proportions in both groups, with a slight predominance of headaches and/or migraines and behavioural disorders in adults. After a year on the GFD a significant improvement was observed in most of these symptoms and signs, both in children and adults, similar to what generally occurs in patients with NCGS without associated GTS [8,34,41].

We found a significant improvement in the neurological signs of GTS after one year on the GFD, with a notable reduction in motor and vocal/phonic tics and OCD symptoms, both in children and adults. A probable explanation lies in the presence of an increase in intestinal permeability of patients with NCGS, as happens in coeliac patients. This enables the passage of gluten peptides and other related peptides to the bloodstream, crossing the blood-brain barrier and reaching different areas of the brain, provoking the appearance of inflammatory processes localised in various structures within the brain, which might explain the presence of the symptoms and signs related to the GTS [34]. This would explain why the withdrawal of gluten from the diet produces a reduction in such deposits and thereby gives rise to significant clinical improvement in the evolution of motor and vocal/phonic tics and OCD symptoms. As Hadjivassiliou stated more than 15 years ago, “Gluten sensitivity can be primarily and at times exclusively a neurological disease. The absence of an enteropathy should not preclude patients from treatment with a gluten-free diet. Early diagnosis and removal of the trigger factor by the introduction of gluten-free diet is a promising therapeutic intervention” and consequently the fact “that gluten sensitivity is regarded as principally a disease of the small bowel is a historical misconception” [42,43,44].

The GFD produced a clear improvement in generic and specific quality of life in both groups, accompanied by a reduction in the overall consumption of drugs, this being more pronounced in adults than children, largely due to the notable reduction in the consumption of psychotropics in the former group, but not significant.

The improvement found with respect to the presence of tics was maintained upon evaluating the degree of disability generated for the motor and vocal/phonic tics, although it was somewhat higher in the children than the adults.

As confirmed by a systematic review, the risk of developing neurological complications in coeliac patients is lower in children than in adults and their response to a GFD is generally quicker and stronger, probably because they have spent less of their life eating gluten [45,46].

Likewise, an improvement was observed in the disability related to the presence of OCD, which is also more striking in children than in adults. We have only found two previous reports in the literature, one of a patient with OCD associated with GTS, and another of an isolated case, both of which showed an improvement in symptoms along with a reduction in their previous disability [17,18].

The patients with GTS, as well as presenting motor and phonic tics, may develop multiple behavioural problems in response to the impact of the symptoms that affect their relationships with family members, friends, class-mates and teachers. Furthermore, it has been estimated that around 90% present other comorbidities, including, amongst others, OCD and those related to ADHD, which exacerbate the disorders of character and behaviour they already had that arose from the presence of tics [47,48].

We can conclude that the improvement of the patients cannot be justified solely by the passage of time because the children were in the stage of worse evolution of the GTS and the adults belong to the subgroup of people whose disorder does not ameliorate or even get worse. In addition, the follow-up period was only a year and the majority of patients had associated comorbidities. In the evaluation and follow-up of diet compliance, 22 of the 29 patients indicated that they had suffered clearly identified occasional contaminations due to errors in their diet. The tics reappeared or worsened in all cases (16 cases with phonic and motor tics, 4 cases with only motor tics, 2 cases with only phonic tics); all of the cases who previously had comorbid OCD experienced its reappearance or intensification. The exacerbation was resolved after days or even weeks of resetting the gluten-free diet. It is interesting to note that since these are inadvertent and involuntary contaminations verified *a* *posteriori*, mainly associated with misinterpretations of labelling, eating out at restaurants or in family homes, the nocebo effect can also be ruled out, especially in the case of children because they do not know the detailed information about the diet. These data indicate a clear relationship between the improvement of GTS symptoms and the withdrawal of gluten from the diet that is not conditioned by the passage of time or hypothetical spontaneous remission.

We evaluated the symptoms related to the tics and the OCD using questionnaires that are widely validated and accepted internationally. However, we did not evaluate the symptoms related to ADHD, although we also found that they improved while on the GFD, as has been confirmed in a recent systematic review of this subject [49].

This paper presents the results of a prospective uncontrolled cohort study, designed as a pilot, and is the first of its kind, as far as we know. It has certain limitations, since the sample size of the study was small, especially in the group of adults, and we have not been able to include a control group. Our initial intention was to include it, but this was not possible. The patients who contacted us presented significant affectation of their quality of life and all of them wanted to try the diet. This prevents us from drawing definitive conclusions, added the fact that we cannot be sure that either the children or the adults followed the GFD fully. Gluten is ubiquitous and removing it strictly from the diet is difficult, especially when eating outside the home [50]. Ensuring fulfilment of the GFD is complicated, as other authors have found [20,50,51,52,53] and we cannot be certain that it was achieved in this study. Current studies show that compliance with the diet in patients with gluten sensitivity is much worse than was formerly considered, demonstrating that approximately 79% of them continue to present intestinal lesions, despite maintaining treatment with the GFD [52]. None of the methods used to evaluate the strict compliance of the GFD has proved to be sufficiently accurate: questionnaires filled in by patients; evaluation of symptoms; determination of gluten-specific antibodies; and findings in duodenal biopsies [51,54]. Frequently, people with a poor educational level and a poor understanding of how to follow a GFD believe that they are strictly following the diet, when in fact they are frequently making mistakes [50,52]. This leads patients to overestimate their compliance when they fill in the questionnaire, making their results unreliable. Neither the absence of digestive symptoms nor the negativity of the antibodies guarantees that the intestinal mucosa recovers, which is complicated to determine with biopsies because the intestinal lesions usually consist of minimal changes without villous atrophy, and that are frequently patched and difficult to identify [51,54,55]. Recently, new methods have been developed to monitor strict adherence to the diet, based on the determination of the presence of gluten peptides in faeces or urine, which seem to offer a realistic alternative, but have not yet been validated or become available for use in daily clinical practice [55]. We gave detailed information to the patients about how to comply fully with the GFD. Although in the case of the children we always recommend to parents that the whole family adopt a GFD, avoiding the consumption of foodstuffs containing gluten at home, in school canteens and elsewhere cannot always be fully achieved. We used questionnaires filled in by the patients (in the case of adults) or their parents (in the case of children) and had regular contact by telephone, e-mail or, in some cases, face-to-face consultation, to monitor compliance and clarify doubts. However, for all the reasons stated above, we conclude that we cannot guarantee that compliance with the gluten-free diet was entirely strict.

## 5. Conclusions

In conclusion, we have shown that following a GFD opens up a new line of therapy for patients with GTS. It is entirely innocuous but requires a strict and prolonged adherence. It seems to be useful for reducing the frequency and intensity of motor and vocal/phonic tics, and OCD symptoms. It is also accompanied by an improved quality of life, both generally and specifically, and a reduction in the consumption of NSAID drugs by children and of antipsychotics by adults.

Subsequent controlled and/or multi-centre studies including more patients and with a prolonged period on the GFD will enable the efficacy of the diet to be determined more exactly.

## Figures and Tables

**Table 1:** Baseline demographic characteristics of children and adults.

Parameters	Children (*n* = 23)	Adults (*n* = 6)	*p*
Males, *n* (%)	17 (74)	3 (50)	NS
Mean age, years, (*X* ± SD)	8.3 ± 2.7	32.2 ± 11.9	NA
Age at commencement, years, (*X* ± SD)	3.8 ± 2.0	7.7 ± 3.4	NA
Duration of symptoms of GTS, years, (*X* ± SD)	4.5 ± 2.4	24.5 ± 10.9	NA
Associated OCD, *n* (%)	16 (69)	6 (100)	NS
Associated ADHD, *n* (%)	11 (52.2)	4 (66.2)	NS
Total YGTSS score, (*X* ± SD)	55.0 ± 17.5	55.8 ± 19.8	NS
Total Y-BOCS score, (*X* ± SD)	15.3 ± 12.3	26.8 ± 9.2	=0.043
Total EQ-5D score, (*X* ± SD)	0.6 ± 0.2	0.5 ± 0.2	NS
Total GTS-QOL score, (*X* ± SD)	42.8 ± 18.5	64.0 ± 7.9	=0.000
Drug consumption, *n* (%)	21 (91.3)	6 (100)	NS
-NSAIDs, *n* (%)	21 (91.3)	6 (100)	NS
-Psychotropics, *n* (%)	8 (34.8)	6 (100)	=0.006

The Mann Whitney or Fisher tests were used when the variances were different and when they were similar, the Student test was employed. *X* = Mean; SD = Standard Deviation; OCD = Obsessive Compulsive Disorder; ADHD = Attention-Deficit/Hyperactivity Disorder; YGTSS = Yale Global Tics Severity Scale; Y-BOCS = Yale-Brown Obsessive-Compulsive Scale; EQ-5D = EuroQol-5D; GTS-QOL = Gilles de la Tourette Syndrome-Quality of Life Scale; NSAIDs = Non-Steroidal Anti-Inflammatory Drugs; NS = Non-Significant; NA = Not Applicable.

**Table 2:** Baseline characteristics of the symptoms and signs of NCGS in children and adults.

Parameters	Children (*n* = 23)	Adults (*n* = 6)	*p*
Upper respiratory tract infections, *n* (%)	20 (86.9)	4 (66.7)	NS
Lower respiratory tract infections, *n* (%)	15 (65.2)	2 (33.3)	NS
Associated allergies, *n* (%)	12 (52.2)	2 (33.3)	NS
Headaches and/or migraines, *n* (%)	11 (47.8)	5 (83.3)	NS *
Infectious oral processes, *n* (%)	20 (86.9)	4 (66.7)	NS
Other dental changes, *n* (%)	18 (78.3)	6 (100.0)	NS
Musculoskeletal affectation, *n* (%)	21 (91.3)	5 (83.3)	NS
Dermatitis, *n* (%)	20 (86.9)	5 (83.3)	NS
Anaemia and/or ferropaenia, *n* (%)	17 (73.9)	5 (83.3)	NS
Sleep disorders, *n* (%)	21 (91.3)	6 (100.0)	NS
Behavioural disorders, *n* (%)	22 (95.6)	6 (100.0)	NS **
Urinary disorders, *n* (%)	13 (56.5)	4 (66.7)	NS
Dietary disorders, *n* (%)	20 (86.9)	6 (100.0)	NS
Change in intestinal habit, *n* (%)	22 (95.6)	6 (100.0)	NS
Change in stool consistency, *n* (%)	11 (47.8)	1 (16.7)	NS

The Mann Whitney or Fisher tests were used when the variances were different and when they were similar, the Student test was employed. * Comparing intensity, Mann-Whitney U, *p* = 0.1; ** Comparing intensity, Mann-Whitney, *p* = 0.001 NCGS = Non-Coeliac Gluten Sensitivity; *n* = number; NS = Non-Significant.

**Table 3:** Evolution of neurological symptoms and quality of life after 1 year of a GFD.

Parameters	Pre-GFD	Post-GFD	*p*
Total YGTSS score, (*X* ± SD), (Maximum 100)			
-Children	55.0 ± 17.5	27.3 ± 22.3	=0.000
-Adults	55.8 ± 19.8	20.7 ± 13.5	=0.001
Total Y-BOCS/CY-BOCS score, (*X* ± SD), (Maximum 40)			
-Children	15.3 ± 12.3	5.4 ± 8.6	=0.000
-Adults	26.8 ± 9.2	8.0 ± 8.9	=0.001
Total EQ-5D score, (*X* ± SD), (Maximum 1)			
-Children	0.62 ± 0.23	0.88 ± 0.17	=0.000
-Adults	0.50 ± 0.22	0.87 ± 015	=0.004
Total GTS-QOL score, (*X* ± SD), (Maximum 108)			
-Children	42.8 ± 18.5	22.4 ± 19.9	=0.000
-Adults	64.0 ± 7.9	20.5 ± 12.2	=0.001
Drug consumption *			
-Children: -Total consumption, *n* (%)	21 (91.3)	16 (69.6)	=0.001
-NSAIDs, *n* (%)	21 (91.3)	12 (52.2)	=0.002
-Psychotropics, *n* (%)	8 (34.8)	7 (30.4)	=0.190
-Adults: -Total consumption, *n* (%)	6 (100)	6 (100)	=0.072
-NSAIDs, *n* (%)	6 (100)	5 (83.3)	=0.100
-Psychotropics, *n* (%)	6 (100)	4 (66.7)	=0.071

The tests used were the Wilcoxon signed-rank test for adults, the Student paired test for children and the McNemar test for drugs consumption. * Comparing intensity, Wilcoxon test, GFD = Gluten-free diet; X = Mean; SD = Standard Deviation; YGTSS = Yale Global Tics Severity Scale; Y-BOCS = Yale-Brown Obsessive-Compulsive Scale; EQ-5D = EuroQol-5D; GTS-QOL = Gilles de la Tourette Syndrome-Quality of Life Scale; NSAIDs = Non-Steroidal Anti-Inflammatory Drugs.

**Table 4:** Evolution after 1 year of a GFD of the various components of motor and phonic tics, obsessions and compulsions.

Parameters	Pre-GFD	Post-GFD	*p*
Evaluation of motor tics (number, frequency, intensity, complexity and interference), (*X* ± SD), (Maximum 25)			
-Children	18.7 ± 4.3	10.1 ± 6.3	=0.000
-Adults	17.3 ± 7.1	9.0 ± 4.7	=0.027
Evaluation of phonic tics (number, frequency, intensity, complexity and interference), (*X* ± SD), (Maximum 25)			
-Children	14.4 ± 5.6	7.4 ± 7.1	=0.001
-Adults	15.2 ± 5.8	5.8 ± 3.9	=0.028
Overall evaluation of motor and phonic tics (number, frequency, intensity, complexity and interference), (*X* ± SD), (Maximum 50)			
-Children	33.1 ± 8.3	17.5 ± 12.1	=0.000
-Adults	32.5 ± 9.5	14.8 ± 7.7	=0.028
Overall disability of motor tics, (*X* ± SD), (Maximum 5)			
-Children	2.3 ± 1.1	1.0 ± 1.1	=0.000
-Adults	2.5 ± 1.6	0.7 ± 0.8	=0.059
Overall disability of phonic tics, (*X* ± SD), (Maximum 5)			
-Children	2.0 ± 1.2	1.0 ± 1.2	=0.001
-Adults	2.2 ± 1.3	0.5 ± 0.8	=0.041
Overall disability of motor and phonic tics, (*X* ± SD), (Maximum 10)			
-Children	4.4 ± 2.0	1.9 ± 2.1	=0.000
-Adults	4.7 ± 2.2	1.2 ± 1.6	=0.027
Evaluation of obsessions (time, interference, discomfort, resistance and control), (*X* ± SD), (Maximum 20)			
-Children	8.7 ± 6.6	3.0 ± 4.7	=0.001
-Adults	13.5 ± 4.4	4.0 ± 4.5	=0.028
Evaluation of compulsions (time, interference, discomfort, resistance and control), (*X* ± SD), (Maximum 20)			
-Children	6.6 ± 6.3	2.4 ± 4.5	=0.008
-Adults	13.3 ± 4.9	4.0 ± 4.3	=0.027

The tests used were the Wilcoxon signed-rank test for adults and the Student paired test for children. GFD = Gluten-free diet; *X* = Mean; SD = Standard Deviation.

**Table 5:** Evolution of symptoms of non-coeliac gluten sensitivity after 1 year of a GFD.

Parameters	Pre-GFD	Post-GFD	*p*
Upper respiratory tract infections, (*X* ± SD), (Maximum 18)			
-Children	3.2 ± 2.1	0.6 ± 0.9	=0.000
-Adults	4.5 ± 4.9	0.2 ± 0.4	=0.680
Lower respiratory tract infections, (*X* ± SD), (Maximum 9)			
-Children	2.1 ± 2.2	0.1 ± 0.4	=0.001
-Adults	1.3 ± 2.1	0.3 ± 0.8	=0.180
Associated allergies, (*X* ± SD), (Maximum 27)			
-Children	1.8 ± 3.4	1.7 ± 2.4	=0.782
-Adults	1.3 ± 2.8	2.2 ± 3.7	=0.285
Headaches and/or migraines, (*X* ± SD), (Maximum 18)			
-Children	1.7 ± 2.7	0.4 ± 0.6	=0.013
-Adults	4.8 ± 4.9	0.8 ± 1.2	=0.068
Infectious oral processes, (*X* ± SD), (Maximum 15)			
-Children	3.0 ± 2.3	1.0 ± 1.3	=0.000
-Adults	3.8 ± 4.2	0.5 ± 0.8	=0.104
Other dental changes, (*X* ± SD), (Maximum 15)			
-Children	2.1 ± 1.6	1.6 ± 1.3	=0.105
-Adults	2.8 ± 2.1	1.8 ± 1.0	=0.276
Musculoskeletal affectation, (*X* ± SD), (Maximum 36)			
-Children	5.0 ± 5.7	1.4 ± 2.5	=0.002
-Adults	9.0 ± 12.7	1.3 ± 2.0	=0.042
Dermatitis, (*X* ± SD), (Maximum 45)			
-Children	4.8 ± 6.3	1.4 ± 1.9	=0.002
-Adults	6.3 ± 4.9	2.0 ± 2.1	=0.058
Anaemia and/or ferropaenia, (*X* ± SD), (Maximum 24)			
-Children	2.8 ± 3.1	0.8 ± 1.4	=0.004
-Adults	4.0 ± 3.6	1.2 ± 1.5	=0.131
Sleep disorders, (*X* ± SD), (Maximum 27)			
-Children	6.2 ± 6.4	1.6 ± 2.0	=0.000
-Adults	8.7 ± 5.3	2.0 ± 1.3	=0.046
Behavioural disorders, (*X* ± SD), (Maximum 24)			
-Children	7.2 ± 5.6	2.6 ± 4.1	=0.002
-Adults	16.0 ± 3.0	3.0 ± 4.5	=0.028
Urinary disorders, (*X* ± SD), (Maximum 21)			
-Children	1.8 ± 2.7	0.7 ± 1.3	=0.082
-Adults	3.3 ± 5.3	0.5 ± 0.8	=0.109
Dietary disorders, (*X* ± SD), (Maximum 45)			
-Children	9.5 ± 9.5	1.3 ± 1.9	=0.000
-Adults	10.5 ± 9.4	1.8 ± 1.2	=0.027
Change in intestinal habit, (*X* ± SD), (Maximum 33)			
-Children	9.1 ± 6.8	3.7 ± 4.8	=0.001
-Adults	7.7 ± 5.2	2.3 ± 2.1	=0.075

The tests used were the Wilcoxon signed-rank test for adults, the Student paired test for children and the McNemar test for drugs consumption. GFD = Gluten-free diet; *X* = Mean; SD = Standard Deviation.

## Supplementary Materials

The following are available online at http://www.mdpi.com/2072-6643/10/5/573/s1, Video S1: A 7 years old child with GTS and OCD-evolution after 1 year of GFD. We include one video of the evolution of an seven-year-old child, recorded before and after 1 year on the GFD, and the scores he obtained. A clear improvement in his symptomatology can be seen. The child was not taking any medication.
